# Scientific Theories vs. Observed Facts

**Published:** 1877-10

**Authors:** 


### ARTICLE II.

### *Scientific Theories vs. Observed Facts.*

One of the most interesting, and by far the most learned paper read at the recent meeting of the American Dental Convention, was one on spectroscopic analysis, by S. Waterman, M. D., of New York City, a physician of ability and culture, with a strong leaning toward the adoption of any conclusions indicated as the result of scientific investigation, and an enthusiast on the subject of the spectroscope. The paper dealt especially with the subject of anæsthesia, or the use of spectrum in demonstrating the action of anæsthetic agents on the blood, as these changes are read by the spectroscope; and, though a long lecture, was listened to with earnest attention, and at its ending the views advanced and conclusions reached made the subject of considerable debate.

The department of the subject which excited the greatest attention on the part of the hearers was the action of nitrous oxide on the blood, and the structural and mor bific changes brought about in that fluid by the inhalation of this gas, the writer taking special pains to establish a theory startlingly new, *viz.*—that the changes produced in the blood by this so universally used and so confidenly trusted to agent were of the most alarming and dangerous character; were of a radical nature, involving vital processes, and causing permanent structural modifications in the constituents of this fluid. These changes were scribed as being in all respects similar to those seen in blood which had passed from a normal to a putrid condition, the hæmato-crystaline being seized by the anæsthetics, oxydation hindered, decarbonization prevented, and, in a word, a train of deleterious conditions instituted which were only hindered from being always fatal by reason of their limited extent. Thus in the most sweeping manner, pronouncing this gas the most dangerous instead, as is ordinarily supposed, the most harmless, of the several agents whose use has been so valued as standing between suffering humanity and the agonies of surgery.

Certainly this is all very startling. If the dentists who from year to year have been occasionally administering nitrous oxide gas to restrain the terrors of a nervous patient, and to save the shock which comes to the bravest of us when compelled to submit to an ordeal we so instinctively shrink from,—if they have all this time been poisoning their patients, it is certainly time they were made aware of it, if not by observation of the after effects of the gas, then by the spectroscope. (We do not refer, except in terms of condemnation, to those who keep the slaughter houses when human teeth are sacrificed by the dealers in porcelain and vulcanite.) To have one's hæmato-crystalline destroyed or rendered useless, by an agent we have been looking on as the representative of harmlessness itself,—to be told that when we give gas we are causing a change in the blood, similar to that which occurs in septicæmia, and that we have killed more or less of the vital fluid, is enough to "give us pause," and if we administer this agent again will depend somewhat upon the faith we have in the spectroscope, or at least in our faith in those who interpret its utterances.

The evidence of the spectroscope, as we understand Dr. Waterman, is this: In normal blood the instrument shows *two* special dark bands; these it is claimed are always present and characteristic. Blood decomposed by putridity shows when similarly tested *one* dark band, always present and characteristic. Blood exposed to the action of nitrous oxide shows a band similar in appearance and in position, to the one seen in the second instance. Now it is fairly to be questioned it seems to us, whether such sweeping conclusions are justified from such data, when we reflect on the newness of spectroscopy, and the narrowness of coming to a definite judgment on evidence which seems to be illy borne out by the observed facts in the case. It is certainly true that in this day the pronounced opinions of men handling scientific instruments are ordinarily accepted as true, and rightly so perhaps,—for the mistakes they make are after a time set right by counter investigation,—until he is a bold layman who utters his doubts; still we can but feel a reluctance to accepting views, which seem to be founded in a theory not borne out by every-day observed facts, and which the evidence of our senses seemingly disproves. There are men who for years have had opportunity of seeing the effects, both immediate and secondary of this gas, men of fair intelligence, of close habits of investigation, and some of them at least, earnest students of pathology, who cannot feel that the blood is poisoned after this manner, and that the danger is as great as is alleged to be shown by the spectrum.

That the spectroscope is one of the most brilliant of modern instrumental means of scientific research, that its usefulness in scientific investigation is great, is an unquestioned fact; that it has thrown light upon dark and hitherto unsolved problems, and aided in the development of scientific industry is a fact all are ready to concede; but it does seem to us that because an instrument will show sodium, hydrogen, carbon, &c., under combustion, and that will demonstrate many of the hitherto undemonstrated problems of modern chemistry—that because of this it may be implicitly relied on to prove that identical bands in the spectrum prove identical conditions of so complex a substance as the blood,—that it can be an infallible test that blood acted on by nitrous oxide gas and putrid blood are in the same condition, and that such blood in the circulation of an anæsthetised person is decomposed and irrecoverably so, is a conclusion we are reluctant to accept without further proof.

It is one of the incidental misfortunes of science that her devotees are enthusiasts, and enthusiasts are seldom judicial. The man who falls in love with a microscope, or telescope or spectroscope, though earnest in searching after truth, yet may not construe that truth correctly. The disputes on subjects into which chemistry enter are full of illustration of this failing of human nature. Here, in a field where analysis and synthesis, where examination from every stand point is made available, the results are too often contradictory, or in controversy. How many microscopists see the same things in the same way? or worse still, how often they only see that which they are looking for. We have before us, and publish elsewhere an abstract of an article to be found in the Popular Science Monthly, which shows how conscientiously men of acknowledged ability may differ. [See Article——] a scientist is now the less a human being with the inherent frailties.

The facts are, setting aside as valueless the testimony of the ghouls who mutilate their, for the time, dead subjects, whose shops for the extraction by wholesale of priceless organs can only be characterised as places of habitual and intentional malpractice, and who are in no way worthy of a place or name among professional men,—aside from their testimony—and certainly few dead are carried out from their doors, though thousands living enter,—there are, as was stated previously, not a few men of acknowledged ability, of reputation, and of conscientious devotion of duty, who have watched the effects of anæsthetics administered at their hands for years, men whose facilities for seeing the after effects of nitrous oxide are equal to those of the physician in practice, and who have failed to see its toxic effect or pernicious tendenices, who have not detected those slowly creeping and obscure pathological changes which are predicted as liable to take place, and which are seen by some who—say they have seen them.

Men die daily from causes to our feeble understandings greatly insufficient. "Found dead in bed," "dropped dead in the street," "died suddenly whilst conversing with his family,"—and all this without shock which would seem sufficient to fatally disturb heart or brain or lungs. But when the surgeon gives chloroform with a fatal result, death is always charged to theanæsthetic—is this fair?

The truth seems to be that we are much in the dark as to the method of the action of anæsthetics. This is not written in any spirit of deprecation of the value of scientific research in this matter,—it is probable, nay certain that what we may know hereafter will be due to research conducted strictly after methods indicated by science, for science is truth. But it cannot be best always to blindly accept the saying of the scientist because he is a scientist; here, as elsewhere, we must compare the theory with the known facts. Anæsthetics are too frequently administered, and probably the error is in that direction more than in the choice of the agent. It is to be hoped that one result of this discussion will be to check this recklessness of dentists, who with a little firmness and kindness could often dispense with their use, and whose conservative skill should make their use, and the use of the forceps much less frequent than at present. If however, dentists feel that they cannot dispense with nitrous oxide, we are sure that they ought not to waste valuable time in generating it, while a better article than they can possibly manufacture is purchasable. In these days when so much is expected of the dentist—so much general knowledge, so much reading to be done, and if he feels called to do it, so much practical study in microscopy, or spectroscopy even, for he will be none the worse dentist for spending his leisure this way,—when all this is before us, no one should waste his time in making anything of use in his work if he can get it better made by paying for it.

